# Ti_3_C_2_T_*x*_ MXenes as Anodes for Sodium-Ion Batteries: the In Situ Comprehension of the Electrode Reaction

**DOI:** 10.1021/acsaem.4c02777

**Published:** 2025-02-10

**Authors:** Antonio Gentile, Nicolò Pianta, Martina Fracchia, Simone Pollastri, Chiara Ferrara, Stefano Marchionna, Giuliana Aquilanti, Sergio Tosoni, Paolo Ghigna, Riccardo Ruffo

**Affiliations:** † Ricerca sul Sistema Energetico, RSE S.p.A., Via R. Rubattino 54, Milano 20134, Italy; ‡ Department of Materials Science, University Milano Bicocca, via Cozzi 55, 20125 Milano, Italy; § Dipartimento di Chimica, Università degli studi di Pavia, via Taramelli 9, 27100 Pavia, Italy; ∥ Elettra-Sincrotrone Trieste, Basovizza, 34149 Trieste, Italy; ⊥ INSTM, Consorzio Interuniversitario per la Scienza e Tecnologia dei Materiali, via Giusti 9, I-50121 Firenze, Italy; # National Reference Center for Electrochemical Energy Storage (GISEL), Consorzio Interuniversitario Nazionale per la Scienza e Tecnologia Dei Materiali (INSTM), via Giusti 9, Firenze 50121, Italy

**Keywords:** MXene, Ti_3_C_2_T_*x*_, anode, sodium-ion batteries, operando Raman, operando XAS

## Abstract

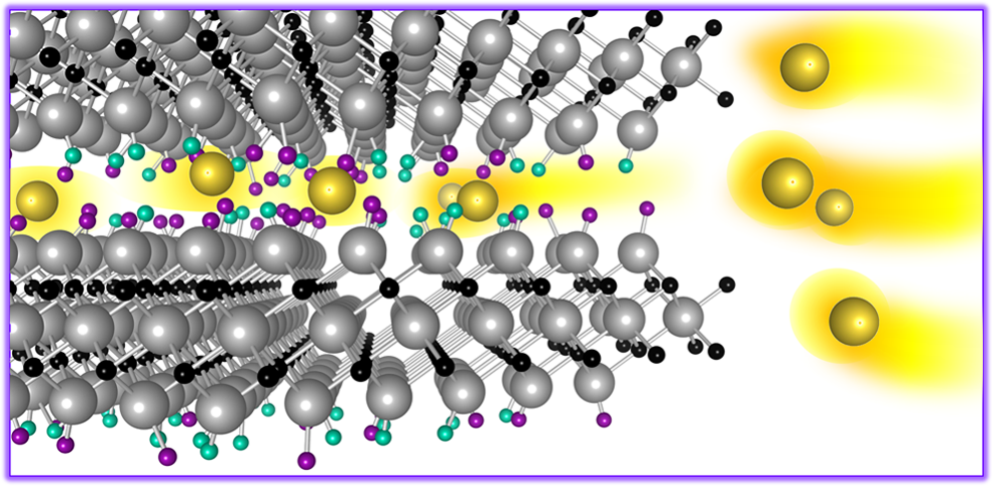

Since their appearance on the scene, MXenes have been recognized as promising anode materials for rechargeable batteries, thanks to the combination of structural and electronic features. The layered structure with a suitable interlayer distance, good electronic conductivity, and moldability in composition makes MXenes exploitable both as active and support materials for the fabrication of nanocomposites providing both capacitive and Faradaic contributions to the final capacity. Although a variety of possibilities has been explored, the fundamental mechanism of the electrode reaction is still hazy. We herein report the investigation of Ti_3_C_2_T_*x*_ MXenes, the benchmark composition for application in energy storage, through the combined operando X-ray absorption spectroscopy (XAS) and Raman analysis supported by density functional theory (DFT) calculations with the aim of clarifying the origin and nature of capacity when the material was cycled vs Na. The electrode reaction determined was Ti_3_C_2_X_2_ + 1Na → Na_1_Ti_3_C_2_X_2_, defining the theoretical capacity.

## Introduction

The MXene class of materials, proposed for the first time in 2011, has quickly become one of the most studied materials for possible application as electrodes in supercapacitors and rechargeable batteries.^1−4^ MXenes are intermetallic compounds with the formula M_a_*X*_c_T_*x*_, with M being the transition metal; X being carbon, nitrogen, or boron; T termination generally being F, O, OH, Cl, or Br.^3,5,6^ While the M and X compositions are determined by the precursor M_a_A_b_*X*_c_ (referred to as the MAX phase, where A is a metal or metalloid element), the T terminations are determined by the etching medium exploited for the selective removal of element A and subsequent functionalization of the MX layers. Although several MXene compositions have been predicted and obtained and new fields of applications are always emerging, the most investigated composition is Ti_3_C_2_T_*x*_, and its use in energy storage devices dominates the fields of applications.^3,7−9^ Indeed, Ti_3_C_2_T_*x*_ can be easily prepared at a laboratory scale and scaled up to industrial levels,^4,10^ as the need for using HF can be circumvented by using less dangerous and sustainable types of etching agents.^1,3,11^ The possible exploitation of Ti_3_C_2_T_*x*_ for ion-storage applications was suggested due to the combination of favorable features such as the layered structure with adequate interlayer spacing, presence of a hydrophilic surface, and high electronic conductivity.^1,12,13^ As a consequence, an impressive number of reports appeared for this composition for applications in rechargeable batteries and supercapacitors.^14−17^

The literature review identified two main strategies for use as electrodes in rechargeable batteries: as bulk active electrode materials or as conductive supporting materials for further functionalization and/or preparation of nanocomposites.^18−26^ The former approach was first explored for Li-, Na-, and K-ion-based batteries; the latter appeared afterward, overcoming the number of combinations and results.^8,27−29^ Although the second possibility leads to larger capacity values, mechanical instability of the composites and the resulting low charging efficiency (≤99%) limit the practical application of these electrodes.^18^

The direct use of MXene as an active material remains of high interest, even providing lower specific capacities, as it is particularly suitable for use in high-power devices, also due to its excellent performance in terms of high charge efficiency and high capacity retention. In addition, the risk of alkali metal plating on the electrode is virtually eliminated due to the high intercalation potential value, achieving high cathodic current and reducing the charging times of the devices.^2,30,31^ Moreover, at a more fundamental level, the understanding of the storage mechanism and electrode reaction is the first step for further developing and optimizing such materials and fully exploiting their potential in energy storage applications. For these reasons, we herein propose a comprehensive investigation of the Ti_3_C_2_T_*x*_ material, the most common and exploited MXene composition as the electrode for rechargeable batteries, with the aim of identifying the electrode reaction mechanism and defining the maximum storage capacity. The investigation was carried out through coupling density functional theory (DFT) calculations with in situ Raman and X-ray absorption spectroscopy (XAS) investigations and finally rationalizing the results with the electrochemical testing. With this approach, we were finally able to provide the theoretical capacity and define the structural variation upon sodiation/desodiation.

## Experimental Section

### Synthesis

The Ti_3_AlC_2_ MAX phase was prepared via the spark plasma sintering (SPS) process. The starting mixture of Ti/Al/TiC powders in a 1:1:1.9 molar ratio was milled in a turbula shaker for 24 h. The obtained precursor was treated at 1300 °C for 5 min considering the heating rate of 80 °C/min, 43 MPa compressive load, and 300 mbar of Ar pressure in the SPS chamber. The obtained Ti_3_AlC_2_ disk was ground and sieved (particle size, <50 mm); the final powders were characterized through X-ray diffraction (XRD) analysis.

The MXT MXene with the composition Ti_3_C_2_T_*x*_ was obtained from 500 mg of Ti_3_AlC_2_ powders by leaching with 10 mL of 5% HF (Sigma-Aldrich, HF >40 wt%, CAS 7664-39-3) for 24 h in a Teflon beaker at room temperature for the preparation under mild conditions.

The syntheses of the same MAX phase and MXene compositions have already been reported under the same experimental conditions in our previous work; the results reported for the Scanning electron microscopy–energy dispersive X-ray spectroscopy (SEM–EDX), thermal analysis, and X-ray photoelectron spectroscopy (XPS) investigations were thus considered as reference data for the analysis of the samples in the present study.^11,15,32^ The samples will be indicated in the following as MAX for the Ti_3_AlC_2_ and MXT for the Ti_3_C_2_T_*x*_ compositions obtained with 5% HF.

### X-ray Diffraction

XRD data were collected on a Rigaku MiniFlex 600 in Bragg–Brentano (θ–θ) geometry with acquisition in the 5–80° angular range, with a step size of 0.02°, using Cu Kα radiation. SEM data were acquired using a Zeiss Gemini electron microscope equipped with a field emission source; collected data were analyzed with the ImageJ software

### DFT Calculations

All calculations were performed with the code VASP 6.^33^ The Perdew, Burke, and Ernzerhof (PBE) exchange and correlation functional was adopted.^34^ A basis set of plane waves with a kinetic energy cutoff of 400 eV was used; the cutoff was increased to 600 eV for lattice relaxation. The core–electron interaction was described using the projector augmented wave (PAW) method.^35^ C(2s, 2p), Ti(3p, 3d, 4s), O(2s, 2p), F(2s, 2p), and Na(2p, 3s) electrons were treated explicitly. The long-range dispersion forces were considered using the damped Grimme DFT+D3 approach.^36^ The reciprocal space was sampled with a 4 × 4 × 2 Γ-centered net of *k*-points. Truncation criteria of 10^–5^ eV (electronic loop) and 10^–2^ eV/Å (ionic loop) were adopted for the structure relaxation. Phonon frequencies at Γ were calculated within the harmonic approximation by diagonalization of the Hessian ionic displacement matrix. A step of 0.015 Å along the displacement vectors corresponding to each vibrational normal mode was used. Two displacements per direction were performed. The macroscopic dielectric tensor was calculated using the density functional perturbation theory.^37,38^ Raman intensities were calculated from the change induced in the dielectric tensor by the ionic displacements along the normal modes, using the Python interface developed by Fonari and Stauffer.^39^ The simulated spectra were generated by convoluting the contribution of each individual normal mode. For each mode, a Lorentzian function centered at the calculated frequency was attributed, whose integral was set equal to the calculated intensity. A half-height width of 3 cm^–1^ was empirically attributed to all peaks.

A 2 × 2 supercell of the Ti_3_C_2_ MXene was adopted, with stoichiometry Ti_12_C_8_T_8_, where the etching group T is either oxygen (O-terminal) or fluorine (F-terminal). Sodium intercalation was simulated by placing either two or four Na atoms in the interlayer spacing. This corresponds to a specific capacity in the range between 60 (Na_2_) and 120 (Na_4_) mAh g^–1^. The stacking of the lamellae was simplified by assuming a perfect epitaxial coincidence along the [001] direction.

At first, the ionic positions and lattice parameters were relaxed. Next, the phonon frequencies were calculated for Ti_12_C_8_O_8_, Na_4_/Ti_12_C_8_O_8_, Ti_12_C_8_F_8_, and Na_4_/Ti_12_C_8_F_8_. The complete calculation of the Raman spectrum was performed in one case (Ti_12_C_8_O_8_) only. The most relevant active modes were then identified in the other structure by analyzing the associated ionic displacements.

### X-ray Absorption Spectroscopy

X-ray absorption spectra were acquired at the Ti K-edge (4966 eV) on the XAFS beamline at the Elettra synchrotron radiation facility in Trieste, Italy. For the ex situ spectra of MXT, an appropriate amount of sample, to give unitary edge jump in the absorption coefficient, was mixed with cellulose and pressed into a pellet. The spectra were then acquired in the transmission mode. Analogously, XAS spectra of TiO, Ti_2_O_3_, and TiO_2_ were collected as reference spectra. For the operando experiments, XAS spectra were acquired in fluorescence mode using a silicon drift detector. The ring current and energy were 200 mA and 2.4 GeV, respectively. A Si(111) double-crystal monochromator was used, ensuring high-order harmonic rejection by detuning the second crystal. A water-cooled, Pt-coated silicon mirror was used to obtain the vertical collimation of the beam. XAS spectra were acquired during the charge/discharge process of the cell, each spectrum lasting for approximately 40 min. The operando electrochemical cell was an ECC-Opto-Std test cell (EL-CELL) equipped with a beryllium window for X-ray experiments. XAS spectra were coupled to electrochemical cycling following the protocols described in the Experimental Section. The X-ray signal extraction and analysis was performed by means of Athena, belonging to the set of interactive programs IFEFFIT. For the X-ray absorption near-edge structure (XANES), the spectra were first background-subtracted using a straight line and then normalized to unit absorption above 500 eV after the absorption edge.

Ex situ XAS spectra at the Ti L_2,3_-edges were acquired at the APE-HE beamline at the Elettra Italian synchrotron. For this purpose, MXT electrodes were quenched at three potentials (0.1, 1, and 3 V vs Na) during the first cycle. These samples were mounted in the sample holder in a N_2_-filled glovebox (H_2_O and O_2_ < 0.5 ppm) and moved to the ultrahigh vacuum chamber of the beamline, where the spectra were acquired in the total electron yield mode. The XAS data were analyzed according to the multivariate curve resolution-alternating least squares method; further details can be found in the Supporting Information.

### Raman Spectroscopy

Raman spectra were recorded in a Horiba XploRA PLUS system equipped with an Olympus BX43 microscope and a long working distance 50× (NA = 0.50) objective. Raman measurements were carried out with a 785 nm solid-state laser and 1200 line/mm diffraction grating that, combined with the resolution (1024 × 256 pixels, pixel dimensions: 26 × 26 μm^2^) of an air-cooled Peltier CCD detector, ensure ≈2 cm^–1^ spectral sensitivity. The laser wavelength chosen for these Raman measurements, at the same time, favored the intensity increase of some resonant Raman modes and avoided luminescence phenomena related to chemical species of the electrolyte used in the half cell for the spectro-electrochemical test.^40−42^ The power of laser was kept at ∼5–8 mW both to reduce the local heating of the materials and minimize their degradation/change, and at the same time to collect optimal Raman signals in order to reduce the time step (15 min) between two subsequent spectra. The latter aspect is crucial to follow in the best way the evolution of the signals of Raman modes during cycling and, consequently, obtain a more detailed data pattern from which a refined model was derived to describe the interactions between sodium ions and the electrochemically active sites of the MXenes. During electrochemical cycles, autofocus mode along the *z*-axis was included in the routine before the acquisition of each spectrum. With this method, automatically managed by the software (LabSpec6) of the Horiba system, the potential losses of the Raman signals induced by the vertical shift of the electrode during electrochemical processes can be minimized. The focus of the objective was scanned in the range −3/+3 μm around the z-position set at the beginning of the in operando measurement with a pass of 0.3 μm, searching the z-position for which the maximum of the Raman signal is between 700 and 750 cm^–1^ and the interval that includes the most intense vibrational peak of Ti_3_C_2_T_*x*_ (∼730 cm^–1^, see mode 1, Figure 4a).

### Electrochemical Testing

The full cell was cycled with a Hohsen HS 3E cell. The cell was assembled with a 3-electrode configuration, where Na_3_V_2_(PO_4_)_2_F_3_ (named NVPF) was used as the positive electrode (synthesized using the procedure reported in the previous article), MXT as the negative electrode, and metallic Na as the reference electrode.^43^

NVPF and MXene electrodes were prepared by mixing the active materials, Super P (Alfa Aesar; CAS 1333-86-4), and poly(acrylic acid) (Sigma-Aldrich; CAS 9003-01-4) in a 8:1:1 ratio and mixed for 1 h with a Ultra Turrax T-50 mixer at 1000 rpm using *N*-methyl-2-pyrrolidone as medium. The obtained slurry was cast on a 15 μm thick aluminum foil (MTI), allowing us to obtain a 250 μm thick electrode through the doctor blade technique. The slurry was dried for 12 h under vacuum at 120 °C to fully remove the solvent and subsequently colandered.

To reduce the irreversibility of MXT in the first cycle, the electrode was cycled between 0.1 and 3.0 V vs Na^+^/Na for 5 cycles using the reference electrode as the counter electrode. In the full cell, the potentials of the positive and negative electrodes were measured with respect to the reference electrode. The full cell was cycled using the GCPL2 procedure included in the EC-Lab software (Biologic), allowing control of the cutoff for all of the electrodes. The settings were as follows: E_NVPF vs E_MXT between 0.0 and 5.0 V, MXT vs Na^+^/Na between 0.1 and 3.0 V, and NVPF vs Na^+^/Na between 2.5 and 4.7 V

XAS and Raman in operando measurements were carried out using an EL-CELL ECC-Opto-Std electrochemical cell. The electrode for XAS and Raman operando measurements was always prepared with the same protocol and recipe as a self-standing electrode by dispersing the active material, PVDF, and Super P carbon in a weight ratio of 70:20:10 dissolved in *N*-methyl-2-pyrrolidone, using IKA’s Ultra-Turrax T-50 homogenizer. The mixture was deposited with a thickness of 15 mils, dried in a vacuum oven, and cut into 14 mm round discs.

For XAS measurements, the cell was assembled by using a beryllium optical window; for Raman measurements, a glass optical window was used. The optical cells were assembled using MXene as the active material and sodium metal as the counter electrode, while the electrolyte was a 1 M NaPF_6_ dissolved in a solution of ethylene carbonate (EC) and diethyl carbonate (DEC) at 1/1 %vol. The cells were cycled in the range 3.00 to 0.1 V vs Na^+^/Na by applying a constant current of 15 mA g^–1^.

## Results and Discussion

**Figure 1:**
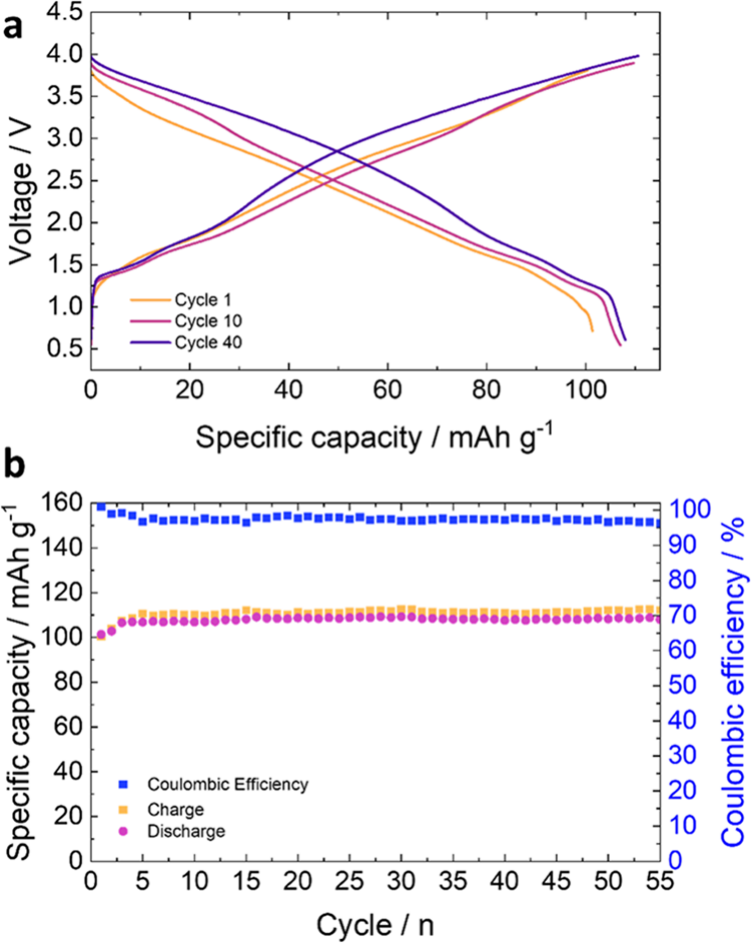
Electrochemical performance of an MXT–NVPF full cell. (a) Potential vs gravimetric charge profile for cycle 1 (orange), 10 (purple), and 40 (violet); (b) specific capacity and Coulombic efficiency as a function of cycles for the first 55 cycles of the cell. For both graphs, the electric charge has been normalized by the mass of the MXene.

The promising electrochemical behavior of MXene-based electrodes is herein demonstrated by the electrochemical results of a full cell assembled with Ti_3_C_2_T_*x*_ (MXT in the following) and Na_3_V_2_(PO_4_)_2_F_3_ (NVPF in the following) as negative and positive electrodes, respectively (Figure 1). The MXT was obtained under mild etching conditions as previously reported; details of materials preparation and structural and morphological characterization and cell assembly are presented in the Supporting Information Section 1 (SI-Sec 1), Figure S1 and SI-Sec 2, Figure S2, respectively, and in our previous works.^11,15,44^ The charge/discharge profiles (Figure 1a) are mainly dominated by the typical linearly sloped behavior of Ti_3_C_2_T_*x*_ (see Figure S2 for profile contributions). Sodium can interact with MXene both through simple adsorption on its surface and by intercalation within its planes.^2,14,30,45−47^ This translates into a system capable of withstanding high currents while maintaining excellent capacities and for high numbers of cycles at the price of a higher average operating potential. Nevertheless, the Ti_3_C_2_T_*x*_–NVPF cell can store around 140 Wh kg^–1^ normalized on the electrodes’ active mass, well in line with other full sodium-ion cells proposed in the literature, maintaining good performance for several cycles, as reported in Figure 1b.^48−52^

The peculiar charge/discharge profiles and the electrochemical behavior of Ti_3_C_2_T_*x*_ depend on the synthesis of the material^11,15^ and the ionic carrier,^15,32^ as demonstrated by our previous works, in agreement with the scenario emerging from the literature.^48,53−55^

Until now, the understanding of the electrode reaction of MXene has been undertaken with different approaches, including DFT,^12,15,31,56^ XPS,^15,49,57^ XAS,^32,58,59^ and XRD,^2,32,59^ but mainly considering ex situ analysis. The picture emerging from this wide literature, however, remains incomplete, and an in operando analysis is still missing. Therefore, we investigated the mechanism implementing operando XAS and Raman experiments coupled with a DFT-based computational approach.

**Figure 2:**
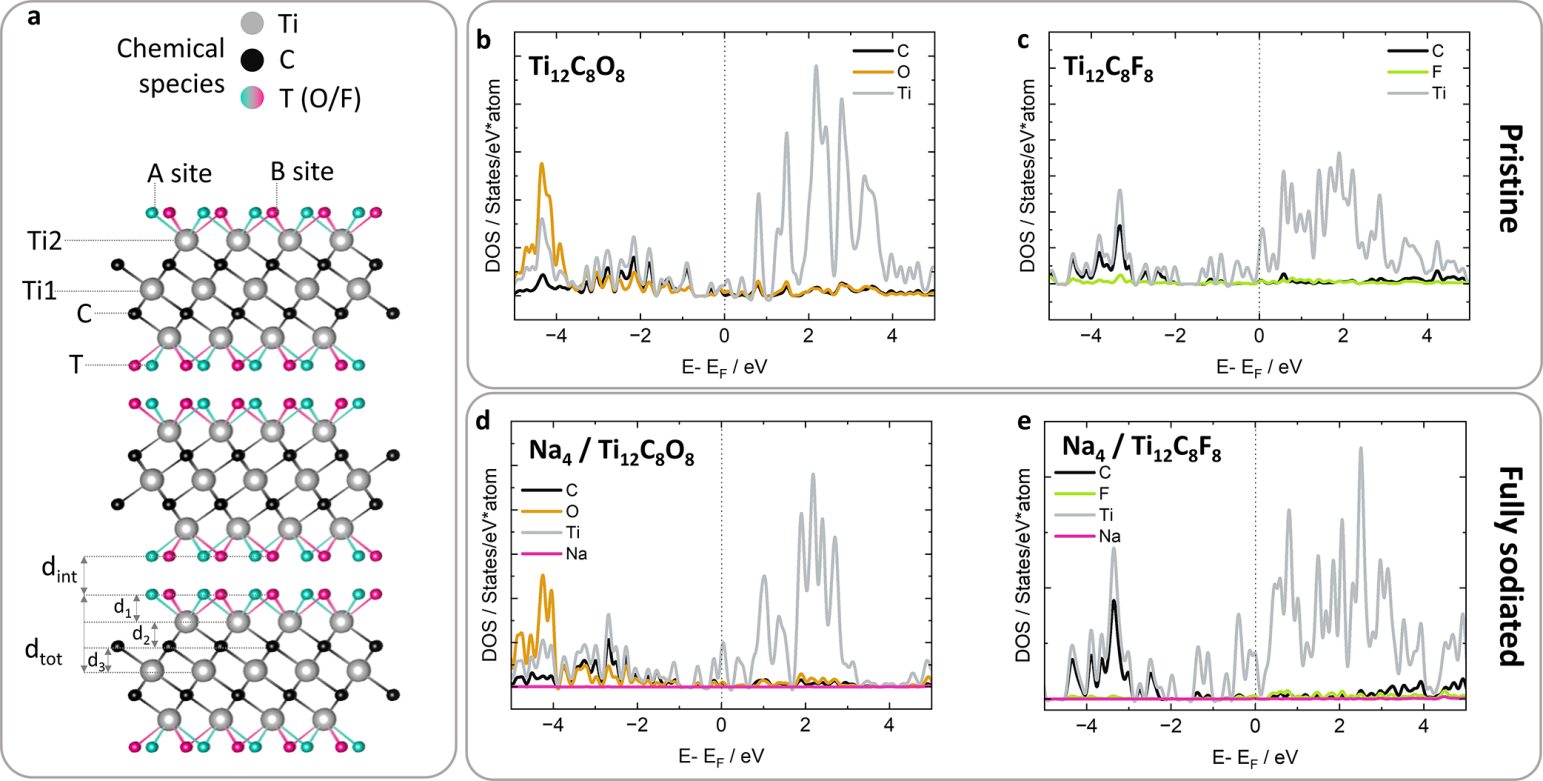
Pristine Ti_3_C_2_T structure with site attribution, labeling the two most favorable termination sites A (green blue) and B (pink), and marking of the most relevant inter- and intralayer distances discussed in the main text (a). PDOS of pristine structures corresponding to Ti_3_C_2_(O/F)_2_ stoichiometry [Ti_12_C_8_O_8_ (b) and Ti_12_C_8_F_8_ (c)] and for the fully sodiated structures corresponding to NaTi_3_C_2_(O/F)_2_ stoichiometry [Na_4_/Ti_12_C_8_O_8_ (d) and Na_4_/Ti_12_C_8_F_8_ (e)].

The first step to comprehend the Ti_3_C_2_T_*x*_ electrode reaction is to assess the electronic structure of the starting compound. The band structure of the MXT materials has been widely investigated through DFT studies, dealing with some preliminary issues as the identification of the termination sites and actual compositions.^1−3,12,49,60,61^ The nature of terminations is indeed a crucial aspect in determining the final electrode performance, and it has been screened deeply by several groups.^15,49,61^ The picture arising from these reports defines the Ti_3_C_2_ layers as highly ordered, while the surface is fully covered with termination groups sitting in the two most favorable sites, A (hcp) and B (fcc), as highlighted in Figure 2a; a detailed discussion can also be found in our previous work.^32^ Exploiting this consolidated knowledge, we here considered the presence of -F and -O terminations on the A and B sites for the building up of the MXene structure; the corresponding electronic structure was calculated as described in SI-Sec 3.

Pristine Ti_3_C_2_ displays a metallic, spin-polarized electronic structure; occupied and virtual states show a predominant component related to the Ti(3d) orbitals in the region near the Fermi level. Introduction of the O- or F-terminal, due to their electron-withdrawing capability, quench the spin imparity in titanium carbide. Nevertheless, both systems considered here (Ti_3_C_2_F_2_ and Ti_3_C_2_O_2_) maintain a metallic character with the predominant Ti(3d) character in the Fermi level region, as evident in the projected density of states. However, a deeper analysis reveals an interesting difference between F- and O-terminated MXT: if a band alignment to a common reference (vacuum level) was performed, Ti_3_C_2_O_2_ shows acceptor states deeper in energy compared to Ti_3_C_2_F_2_, which explains the larger interaction energy of Na^+^ on O-terminated MXenes, as well as the increase in the operational voltage at a lower concentration of fluorine on the MXT surface.^15^

This picture was considered as the starting frame for the introduction of sodium, mimicking the charging process of the negative electrode. Sodium atoms have been introduced in the Ti_12_C_8_T_8_ simulation box in the center of the interlayer distance, allowing the final structure to fully relax; the considered compositions correspond to specific capacity in the range between 60 (Na_2_) and 120 (Na_4_) mAh g^–1^ (details in SI-Sec 3). The structure relaxes slightly, with a modification of the interlayer distance and Na moving from the center of the interlayer through the termination on the top of the central Ti and C atoms (Figure S3 and Table S1), in agreement with previous findings.^30,47,50,52^

A maximum concentration of 4 Na atoms per cell was considered, which corresponded to a full monolayer of Na ions occupying the three-fold hollow sites on the Ti_3_C_2_ surface (Figure S2). Further loading of Na atoms led to the formation of a multilayer Na structure, with a drop in the interaction energy per Na atom as large as 70%. For this reason, the fully sodiated NaTi_3_C_2_T_2_ stoichiometry was evaluated for the calculation of the theoretical capacity, and no higher Na content was considered.

As a consequence of the sodiation process, the electronic structure underwent a significant change due to electron transfer from the Na(3s) orbital to the Ti(e_g_) orbitals. The projected density of states (PDOS) plots (Figure 2b–e) show that the region across the Fermi level displays indeed mostly Ti-character, as already previously reported (see also Figure S4 for a complete overview).^23,30^ The electron transfer from Na- to Ti-orbitals was quantified by using the Bader formalism to estimate the occupation of the Ti-based orbitals. The variation of the average Ti charge ⟨*q*⟩ was calculated as a function of the Na content; results are reported in Table 1, together with data extracted from the electrochemical tests and XAS data discussed below. The effect of the terminating groups on the charge of Ti atoms is quite relevant: larger charges are reported for the O-terminated MXenes, as evident from values in Table 1. Following the sodiation, maximum electron transfers of 0.09 |*e*| (O-terminated MXenes) and 0.23 |*e*| (F-terminated ones) were predicted.

**Table 1:** Average ⟨*q*⟩ Charge of Ti Atoms as a Function of Na Loading as Determined from DFT, Electrochemical Testing, and XAS Measurements^a^

system	⟨*q*⟩ (|*e*|) – DFT	⟨*q*⟩ (|*e*|) – profiles	⟨*q*⟩ (|*e*|) - XAS
Ti_12_C_8_O_8_	+1.68 (Ti1) /+1.92 (Ti2)	0	3.2
Ti_12_C_8_F_8_	+1.58 (Ti1)/+1.74 (Ti2)		
Na_2_Ti_12_C_8_O_8_	+1.65 (Ti1)/+1.86, + 1.92 (Ti2)	60 mAh g^–1^	3.1
Na_2_Ti_12_C_8_F_8_	+1.57 (Ti1)/+1.61, + 1.63 (Ti2)		
Na_4_Ti_12_C_8_O_8_	+1.63 (Ti1)/+1.83 (Ti2)	120 mAh g^–1^	3.0
Na_4_Ti_12_C_8_F_8_	+1.57 (Ti1)/+1.51 (Ti2)		

a Ti1 and Ti2 sites are defined in Figure 2a. The symmetry of the system is reduced for partial Na loading, which leads to different values of Bader charges for the Ti2 species, both reported in the table.

As calculations show the dominant contribution of titanium to the Fermi level region, the Ti K-edge was probed during the sodiation of MXT through XAS acquisition; details can be found in SI-Sec 2 and SI-Sec 4. It is worth noting that F-terminated MXT display a stronger metallic character compared to O-terminated ones, ensuring better conductivity. On the one hand, thus, F-MXT have a smaller theoretical voltage compared to O-MXT, as already discussed in our previous work.^15^

The detailed analysis of the XAS spectrum of Ti_3_C_2_T_*x*_ pristine powder (Figure S6) has already been discussed in our previous work.^32^ Considering a linear dependence between the valence state and the edge-energy position for different Ti references, we could estimate an oxidation state of 3.2 for Ti based on the position of the absorbing edge. Although this attribution is convenient for the following discussion, we here note that it is intrinsically subjected to a certain error, since different ligands (oxygen/fluorine and carbon in this case) may lead to a shift in the edge-energy position.

**Figure 3:**
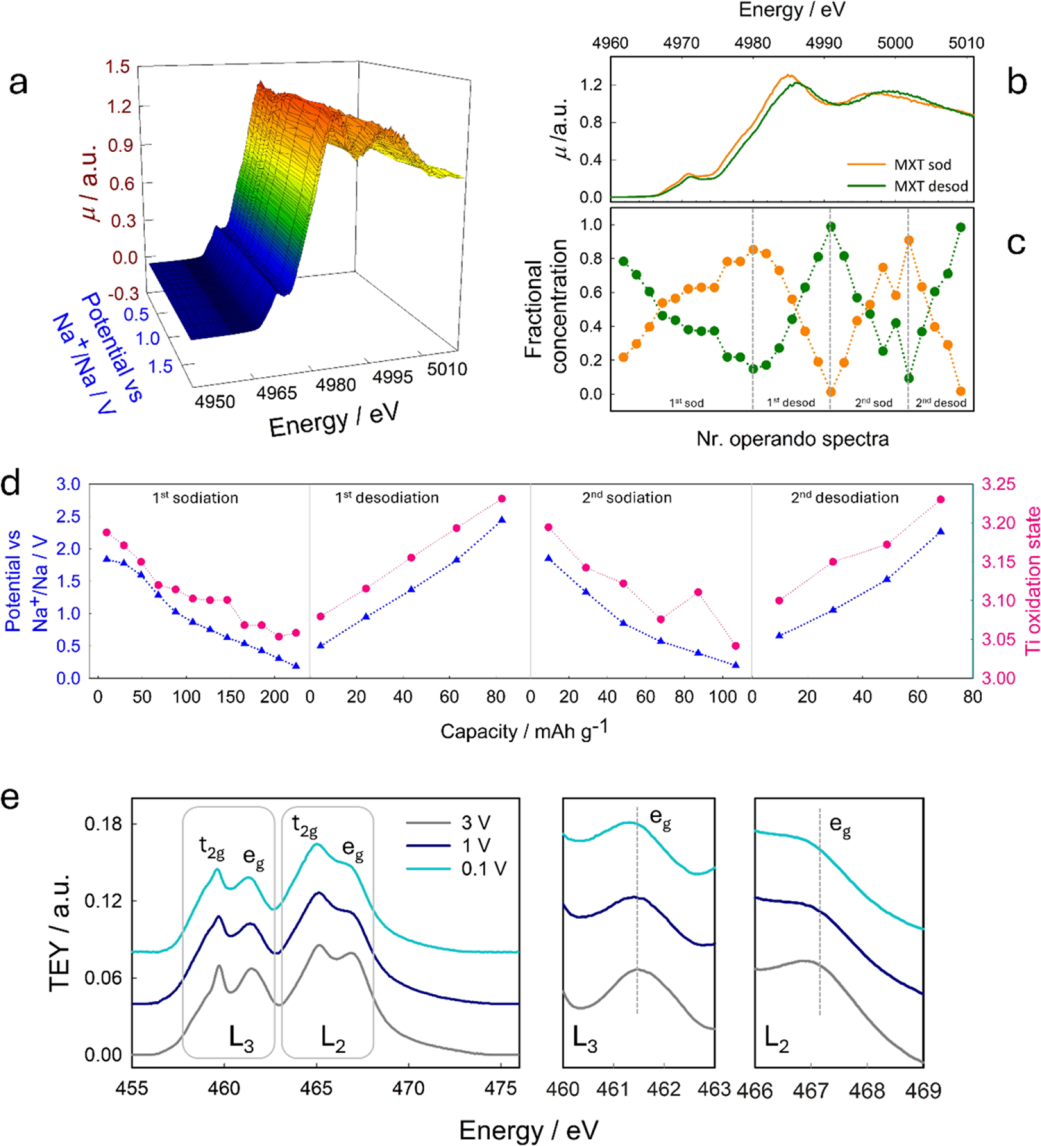
(a) Line plot of the operando XAS spectra (μ in a.u. vs energy in eV) at the Ti K-edge for the Ti_3_C_2_T electrode during the first sodiation process as a function of the explored potential (a); XAS spectra of the independent components determined by the MCR-ALS analysis (b); relative fractional concentration of the two components during first and second electrochemical cycles (c); comparison between voltage profiles explored electrochemically (blue curve) and Ti oxidation state determined from XAS fitting (fuchsia curve) vs capacity for the first and second cycles (d); XAS spectra at the Ti L_2,3_-edges on Ti_3_C_2_T electrodes quenched at 3.0, 1.0, and 0.1 V vs Na^+^/Na (e); the insets provide a magnification of the transitions to e_g_ orbitals.

Operando XANES spectra at the Ti K-edge, acquired during two full electrochemical cycles, are shown in Figures 3 and S7.

Two main trends can be observed with sodiation: (i) the progressive increase of the main peak (white line) intensity and (ii) the shift of the spectra toward lower energies (see the contour plot in Figure S7). The continuous energy shift throughout the potential range indicates that the insertion of Na^+^ in the MXT leads to a progressive increase in the electronic density on Ti. This result supports the mechanism inferred from the electrochemical profiles and the trend outlined by DFT analysis. Moreover, the XAS operando spectra (Figure S7b) highlight a globally reversible behavior. To get more insight into the electrode reaction mechanism, the multivariate curve resolution-alternating least squares (MCR-ALS) strategy combined with principal component analysis (PCA) was exploited to the whole XAS data set; details on the approach and analysis are reported in the SI-Sec 4.^24,62^ It is thus possible to identify two independent and statistically significant components, representing the Ti_3_C_2_T_*x*_ spectrum with the highest content of sodium (orange line) and the spectrum of Ti_3_C_2_T_*x*_ with the lower content of sodium (green line) (Figure 3b,c). The two spectra are shifted of 0.7 eV, from which it is possible to estimate the average Ti oxidation states of 3.0 and 3.2, respectively. The evolution of these two species over the two electrochemical cycles is shown in Figure 3c. Overall, XAS and DFT present a coherent picture of the charge distribution upon sodiation, where the decrease of positive charge on Ti revealed by DFT calculation is complementary to the increase in the electron density inferred from the XAS spectra. The analysis of the XAS spectra reveals an average charge injection close to 0.2 |*e*|, as reported in Table 1. The estimated oxidation state from XAS data at the OCV is close to Ti ^3+^, while Bader charges yield a charge state closer to Ti^2+^. Nevertheless, the oxidation states, as shown by XAS and Bader charge analysis, have different meanings. In the XAS experiments, the edge-energy position was measured and compared to that of standards. Bader charges were computed by partitioning the global electron density on different atoms, and, as a result, often differs remarkably from the attributed formal oxidation states. To quote an example in TiO_2_, where Ti has a formal oxidation state of +4, the Bader charges of Ti atoms are around 2.2 |*e*|.^63^ This reflects the fact that the chemical formalism at the base of the attribution of the oxidation states does not account for the actual nature of the chemical bonds, which, in the case of Ti–C bonds in MXenes, exhibit a nontrivial mixed covalent-metallic nature. Overall, there is satisfactory agreement between experiment and theory, fully supporting the proposed electrode chemical reaction presented here.

Based on this, a direct comparison of the spectroscopic findings with the electrochemical measurement is feasible; results are reported in Figure 3d, where the voltage profiles are plotted together with the average Ti oxidation state vs capacity. The slopes of the two curves are substantially comparable, pointing toward the fact that the MXene is behaving like a pseudocapacitor. The only significant deviation is observed during sodiation in the region between ca. 1.2 and 0.5 V, both in the first and second cycles; this is associated with irreversible processes (e.g., SEI formation).

In order to monitor the Ti 3d state evolution during the electrochemical reaction, we acquired ex situ XANES spectra at the Ti L_2,3_-edges on electrodes quenched at 3.0, 1.0, and 0.1 V vs Na^+^/Na (Figure 3e), details are found in Si-Sec 4. The L_2,3_-edges are due to intense dipole-allowed transitions from the 2p_1/2_ and 2p_3/2_ states, respectively, to empty 3d states. The octahedral crystal field splits the Ti 3d states into 3-fold degenerate *t*_2g_ orbitals and 2-fold degenerate e_g_ orbitals. It can be observed that with a decrease in the potential the intensity of the peaks related to the 2p → 3d *e*_g_ transition decreases significantly. This effect is evident by the insets in Figure 3e and by the calculation of the peak area, reported in Table S3. Only a slight change in the peak shape occurs for the *t*_2g_ orbitals (evident at the L_3_ edge); in this case, the peak intensity remains substantially constant. A decrease in intensity can be attributed to a decreased amount of available empty orbitals, which is expected in the case of a larger electron density on Ti. It can be then stated that upon sodiation electrons are injected directly on *e*_g_ orbitals, in agreement with what is predicted by the DFT calculations.

**Figure 4:**
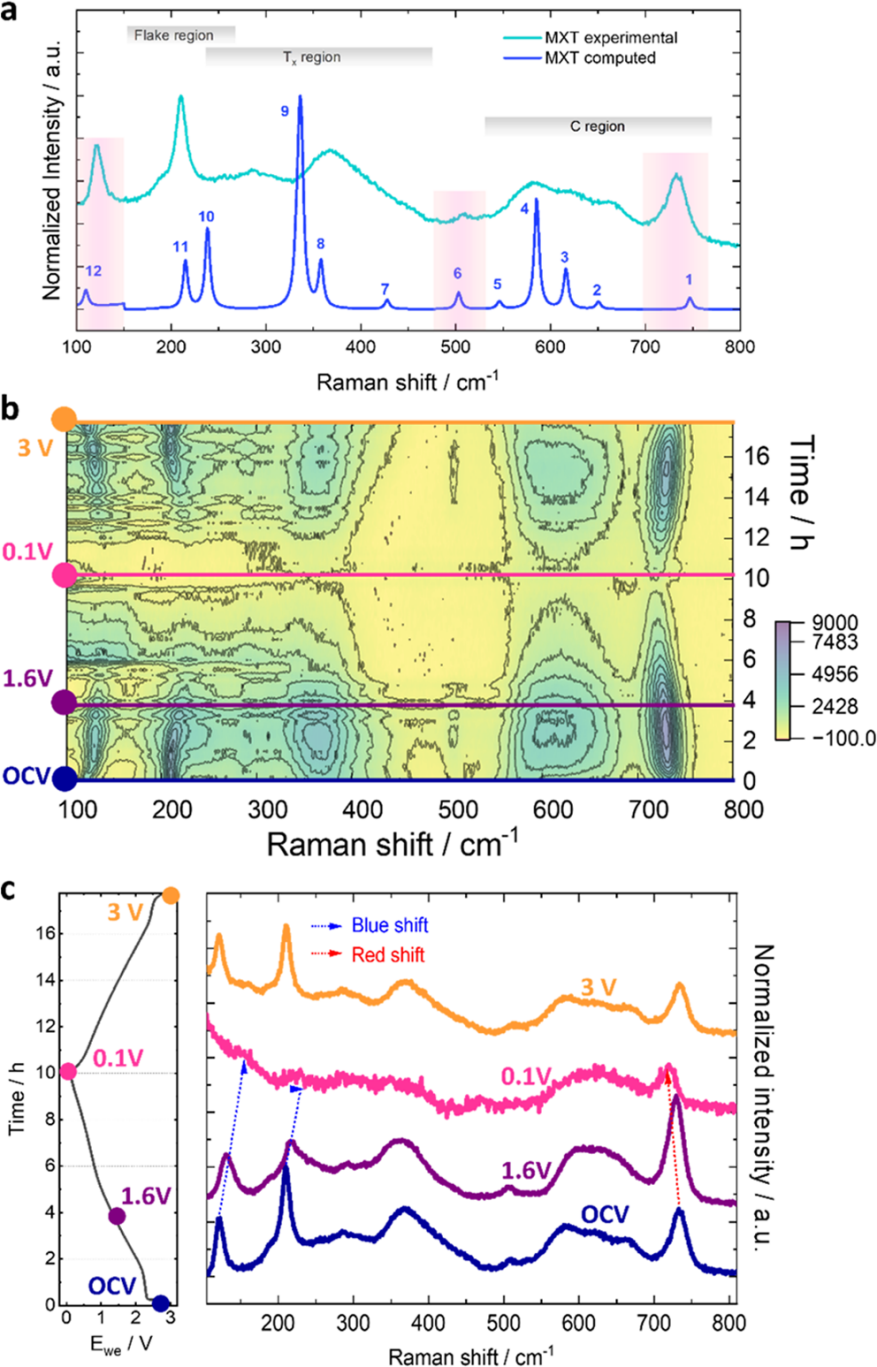
Theoretical and experimental Raman spectra for the pristine MXT sample with attribution for each mode defined in Table S5. The gray strips identify the fingerprint regions, while the pink bars highlight the most significant peaks (a); the complete counter plot of the Raman spectra (after linear background correction) during the first cycle complete from OCV to 0.1 V (sodiation) and return to 3.0 V (desodiation) (b); comparison of Raman spectra selected at specific potential values (colored dot) reported along the galvanostatic cycling with potential limitation (GCPL) profile. The blue and red arrows help to follow, respectively, the reversible blue and red shifts of the most evident Raman signal as a function of (de)sodiation process (c).

The evolution of the electronic structure is closely linked to structural changes in the Ti_3_C_2_T_*x*_ framework during the insertion and removal of sodium. Tracking these changes with XAS analysis is challenging because it cannot distinguish between Ti1 and Ti2 sites. Therefore, we monitored this aspect using operando Raman analysis, applying the same electrochemical protocols used in the XAS experiments. By combining experimental data with computed Raman modes, we were able to assign the different modes accurately with detailed results provided in Figure 4 and in Si-Sec 3 and SI-Sec 5 (Figure S4, and Table S2). It must be recalled that experimentally the MXT sample here considered present mixed O/F terminations with a dominance of oxygen over fluorine, which are previously determined in our previous works.^15,40^ Based on this, the calculation of the Raman modes has been based on the O-terminated MXT structure.

The Raman spectrum of MXenes can be divided into 3 main regions (ray areas in Figure 4a): (i) the flake region, which corresponds to a group vibration of carbon, two titanium layers, and surface groups; (ii) the T_*x*_ region, which includes the vibrational modes of the functional groups on the surface; (iii) the carbon region that collects both in-plane and out-of-plane vibrations of carbon atoms. The simulation of the complete Raman spectrum was performed in the case of Ti_12_C_8_O_8_; the agreement with the experimental spectra of a pristine MXT sample is extremally good (SI-Sec 5).

By inspection of the ionic displacements associated with the active normal modes (Figure S4), it has been possible to evaluate the shift induced upon sodiation, in analogy to the measurements in operando (see Figure 4b). The simulated active Raman modes (Table S2 in Figure S5) reveal a nonbanal picture, where some modes are blue-shifted in the presence of Na cations, while others are red-shifted. A possible interpretation emerges by cross-checking the frequencies, the corresponding ionic displacements (Figure S5), and the structural changes induced by sodiation (Figure S3).

The sodium ions relax moving toward the terminations, thus interacting preferably with a singular MXene lamella instead of coordinating to two adjacent layers, as already reported previously and correlated with the small-ion dimension.^30,47,50,52^ As a consequence, the MXene layer involved in the interaction also relaxes. The simulation of the significant inter- and intralayer distances (defined in Figure 2a) reveals interesting trends (Figure S3 and Table S1). The overall thickness of the lamellae (*d*_tot_) increases remarkably upon sodiation passing from 6.91 to 7.21 Å and from 7.25 to 8.02 Å for O- and F- terminated lamellae. This effect is stronger for F-terminated MXenes, in agreement with previous findings.^64^ The interlayer distance between the terminal groups and the external Ti layer (*d*_1_) increases upon sodiation; again, this effect is more pronounced in the case of F-termination. The vertical *d*_2_ spacing undergoes contraction when full Na coverage is reached. The decrease in *d*_2_, however, is remarkably smaller than the increase observed for *d*_1_. The spacing in the inner region, *d*_3_, finally is not subjected to any significant change.

Thus, the presence of Na cations induces a structural relaxation and an increase in the thickness of the lamellae, in particular, the d_1_ interlayer spacing. This is reflected in the red shift of several normal modes: modes 1 and 3, for instance, mostly related to the vertical displacements of the C atoms, are both red-shifted. Modes 6–9, related to the displacements of the termination groups, are red-shifted as well, as one could expect based on the strong increase in d_1_, as reported in Table S1. Modes 2 and 5, in contrast, display a blue shift, as they are both related to horizontal displacements of the C atom along the *d*_2_ interlayer spacing, which shrinks upon sodiation. The same argument holds true for the blue shift of mode 10, related to the vertical displacement of the Ti ions in the *d*_2_ and *d*_3_ spacing. The blue shift of mode 11, which is an in-phase vertical vibration of all ions in the outer layer, may be due to the physical obstruction represented by the Na cations in the space between the lamellae. These results are in fully accordance with the conclusion derived in the literature based on experimental profiles.^41^ Moreover, the high reversibility of the peak evolution upon cycling (Figure S8) nicely agrees with the XAS observations.

## Conclusions

Based on this unprecedented combined analysis approach, the Ti_3_C_2_X_2_ MXene structure has been deeply investigated from the structural point of view as a pristine material and upon Na-driven reduction, thus allowing us to define the variation induced by the introduction of Na^+^ from the structure and electronic structure points of view. The Ti centers undergo a variation in the formal charge, while from the structural point of view the Ti–C bond angles remain substantially unchanged with only small variations in the bond distances, demonstrating that the Ti–C skeleton is unaltered by the insertion/deinsertion process. The structure relaxes principally through the variation of the Ti-X termination bond length and the interlayer distance, as assessed by DFT and Raman analyses. Na ions are hooked by a termination group, slightly altering the A and B initial positions. Their arrangement into the cell implies that the maximum Na content can be estimated in 1 per unit formula, considering the available terminations. Based on this evaluation, the formal electrode reaction can be proposed as
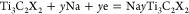
with *y* = 1 as the estimated maximum theoretical capacity. Considering that our MXene has terminations that are a mixture of O and F whose atomic masses are similar, the theoretical specific capacity (*y* = 1) is about 125 mAh g^–1^. This value is comparable to that of oxides such as titania,^65^ titanates,^66^ and nanocomposites based on Sn or Sb^67,68^ but lower than that of hard carbon (300 mAh g^–1^).^69^ The experimentally observed capacity values for the Ti_3_C_2_T_*x*_ material range between 80 and 130 mAh g^–1^; these values have been associated with intercalation chemistry leading to the insertion from 0.5 up to >1 ions per formula unit depending on the termination groups.^12,14,40,45,53,70^ This can be rationalized considering the role of the termination, which affects the average potential, the electronic structure, and the structural relaxation. The operando Raman measurements validated the effectiveness of this technique in both confirming DFT simulation results and providing initial hypotheses about the mechanisms of sodium electrochemical accumulation in the MXene structure. With this in mind, optimizing the synthesis of MXene materials to achieve better control over their terminations could significantly enhance the performance of Ti_3_C_2_T_*x*_ MXene as potential anodes for sodium-ion batteries.

## Supporting Information Available

Pristine material characterization (XRD and SEM), electrochemical testing, computational analysis data, XAS data and discussion, and Raman data and discussion (PDF)

## Abbreviation

GCPL: galvanostatic cycling with potential limitation
