# Supplementary material for: Ti3C2Tx MXenes as Anodes for Sodium-Ion Batteries: the In Situ Comprehension of the Electrode Reaction
Source: ACS Appl Energy Mater. 2025 Feb 10;8(4):2229–38. doi: 10.1021/acsaem.4c02777 (PMC11863289; doi:10.1021/acsaem.4c02777)
Supplement: Supplementary file 1 — ae4c02777_si_001.pdf [file ae4c02777_si_001.pdf]

Supporting Information

$Ti_3C_2T_x$ MXenes as Anode for Sodium-Ion Batteries: the In-Situ Comprehension of the Electrode Reaction

*Antonio Gentile[a], Nicolò Pianta[b], Martina Fracchia[c,e], Simone Pollastri[d], Chiara Ferrara[b,f *], Stefano Marchionna[a], Giuliana Aquilanti[d], Sergio Tosoni[b], Paolo Ghigna[c,e], Riccardo Ruffo[b,f]*

a – Dr. A. Gentile, Dr. S. Marchionna
Ricerca sul Sistema Energetico, RSE S.p.A.,
Via R. Rubattino 54, Milano, 20134, Italy
b – Dr. Nicolò Pianta, Prof. Chiara Ferrara, Prof. Sergio Tosoni, Prof. Riccardo Ruffo
Department of Materials Science
University Milano Bicocca
via Cozzi 55, 20125 Milano, Italy
E-mail: chiara.ferrara@unimib.it
c – Dr. Martina Fracchia, Prof. Paolo Ghigna
Dipartimento di Chimica, Università degli studi di Pavia, via Taramelli 9, 27100, Pavia, Italy;
d – Dr. Giuliana Aquilanti, Dr. Simone Pollastri
Elettra-Sincrotrone Trieste, 34149, Basovizza, Trieste, Italy
e – Dr. Martina Fracchia, Prof. Paolo Ghigna
INSTM, Consorzio Interuniversitario per la Scienza e Tecnologia dei Materiali, via Giusti 9, I-50121 Firenze, Italy
f – Prof. Chiara Ferrara, Prof. Riccardo Ruffo
National Reference Center for Electrochemical Energy Storage (GISEL)
Consorzio Interuniversitario Nazionale per la Scienza e Tecnologia Dei Materiali (INSTM)
via Giusti 9, Firenze, 50121, Italy

Section 1 – Materials preparation and structural characterization

For details on the synthesis of the Ti_3AlC_2 and $Ti_3C_2T_x$ compounds we referred to our previous works on the same materials, hereafter the main information and procedures are reported.[1,2, 3–5,6]

Both the MAX and MXT materials have been successfully prepared as inferred from the XRD patterns reported in Figure S1a. Also SEM images of the MAX and MXT samples present the characteristic accordion-like morphology, in good agreement with those previously reported by our group and previous literature. [1,2,7–10]

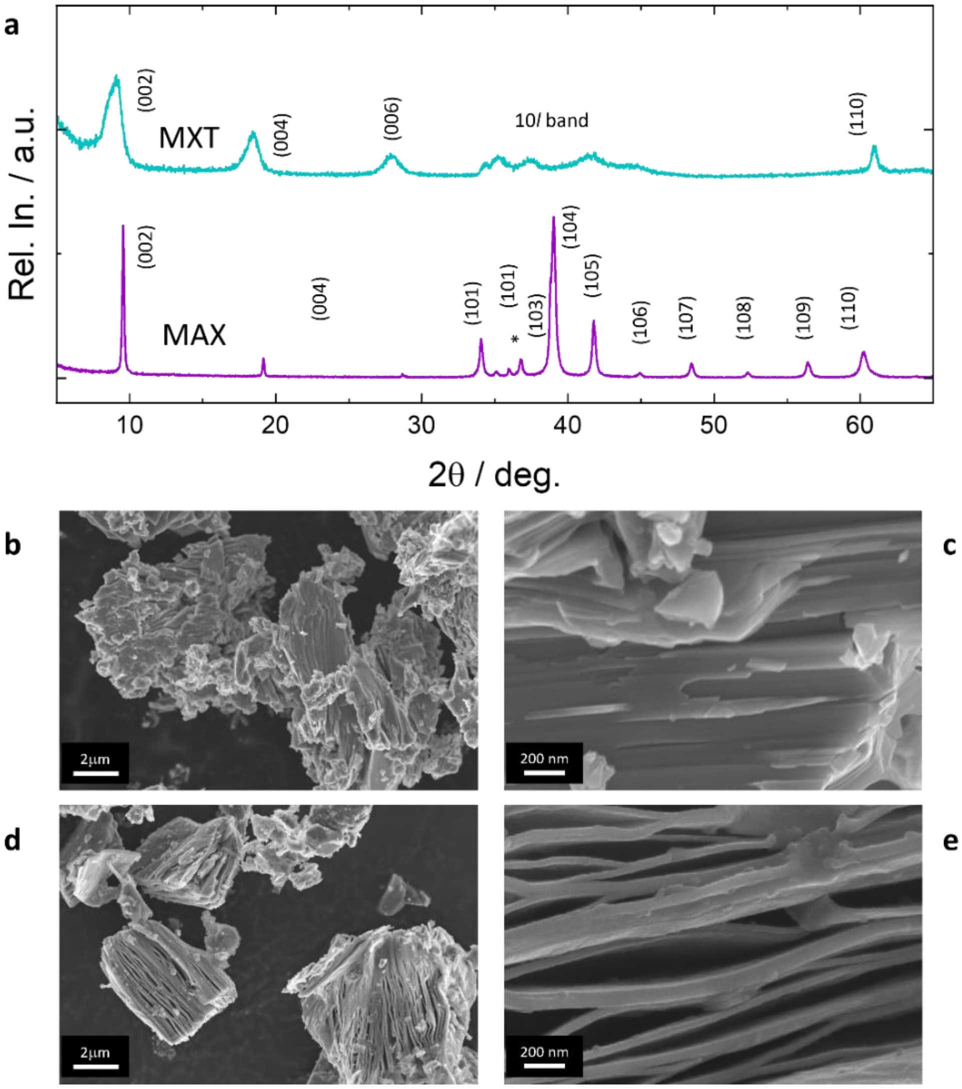

Figure S1. XRD patterns for the pristine MAX phase and for the MXT sample. The * symbol marks the visible reflection of TiC impurity (a); SEM images at different magnification for the MAX sample (b,c) and the MXT sample (d,e).

Section 2 – Electrochemical testing

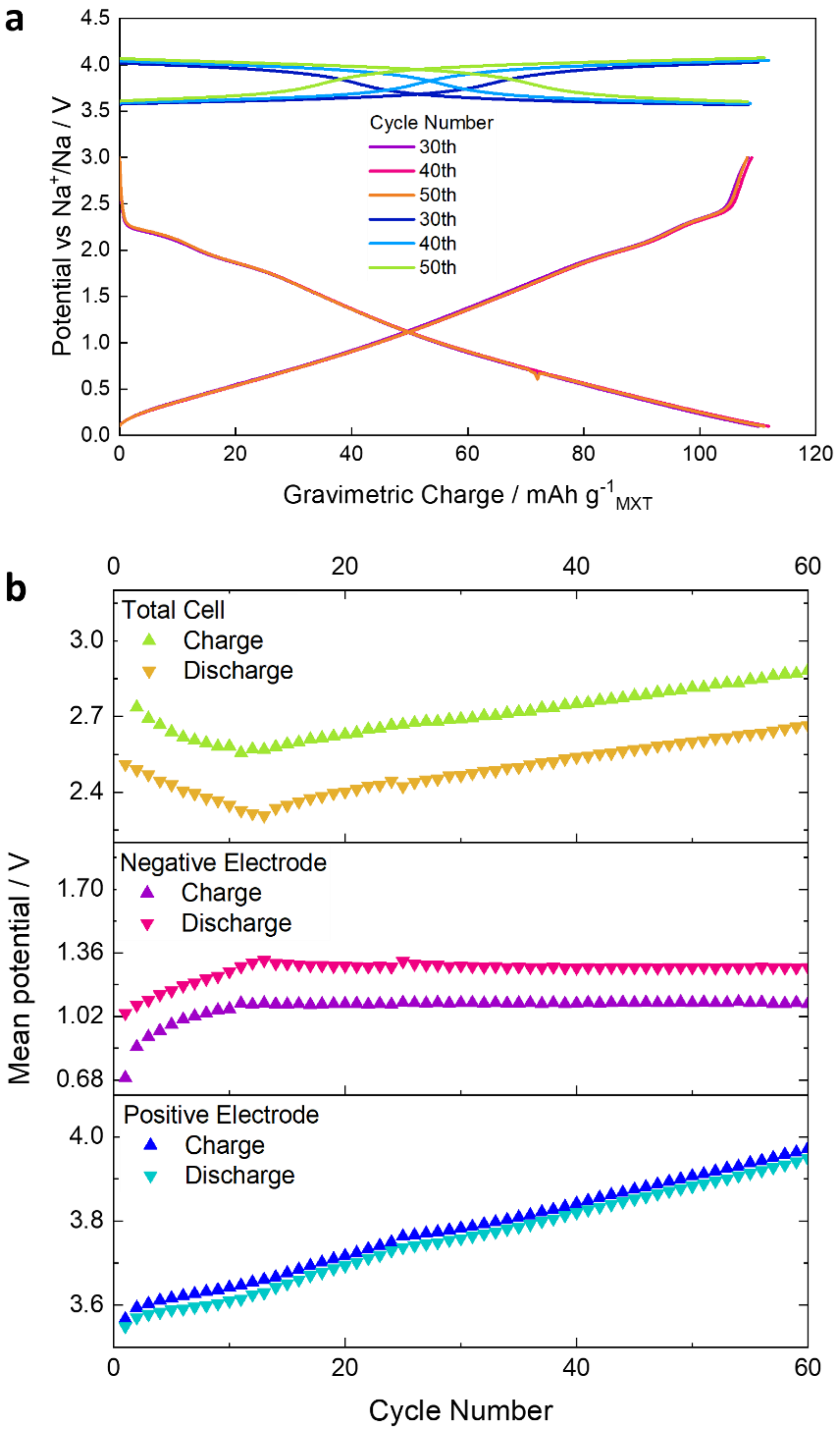

Figure S2. Charge/discharge profiles for the positive and negative electrode for the cycle 30, 40, 50 (a); mean voltage for the full cell, positive, and negative electrodes (b) of the full cell presented in Figure 1 in the main text.[11]

Section 3 – Computational analysis

The details of the calculations are presented in the Experimental section an reference therein. [12-18]

Table S1. Interlayer distances in O- and F-terminated MXenes.

Structure	d_{tot} / Å	d_1 / Å	d_2 / Å	d_3 / Å	d_{int} / Å
$C_8Ti_{12}O_8$	6.91	0.91	1.30	1.24	2.92
$Na_4/C_8Ti_{12}O_8$	7.21	1.18	1.17	1.26	3.24
$C_8Ti_{12}F_8$	7.25	1.26	1.08	1.29	2.87
$Na_4/C_8Ti_{12}F_8$	8.02	1.72	0.99	1.31	2.91

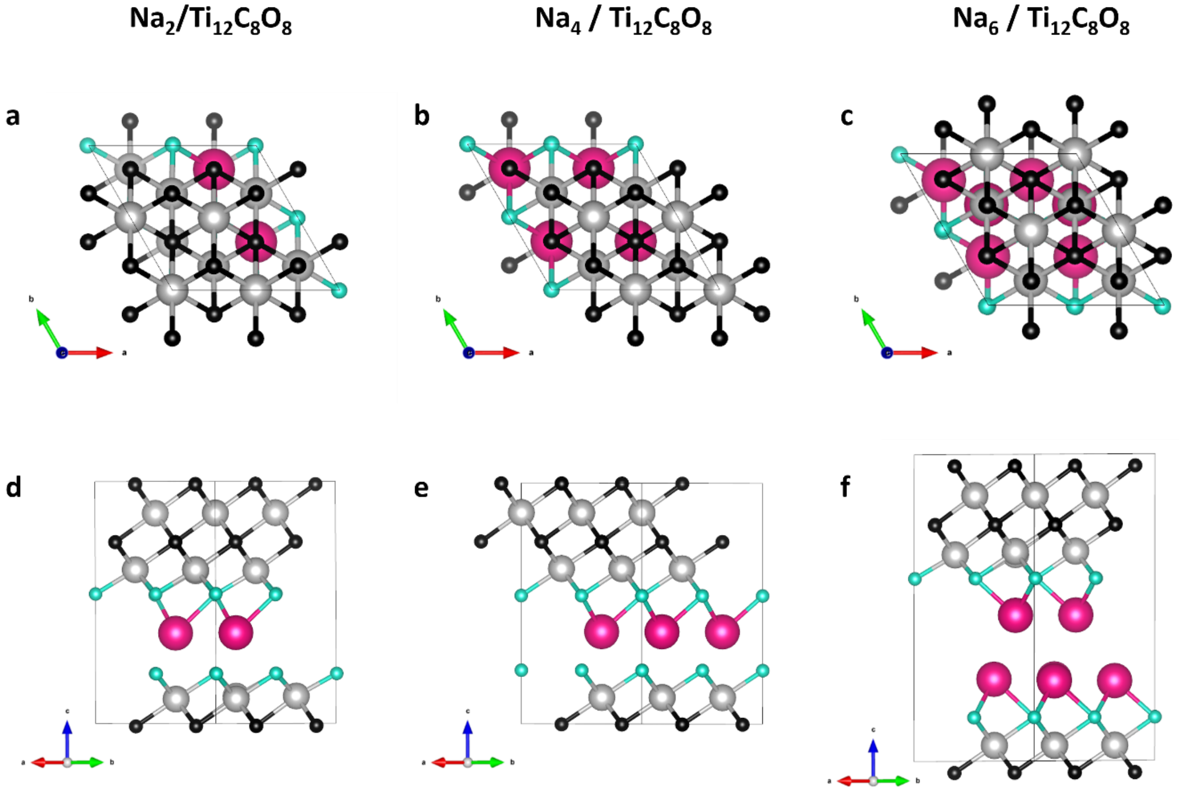

Figure S3. View along c axis (a, b, c) and along ab plane (d, e, f) for the $Na_2/C_8Ti_{12}F_8$ (a, d), $Na_4/C_8Ti_{12}F_8$ (b, e), $Na_6/C_8Ti_{12}F_8$ (c, f) compositions obtained after complete relaxation of the structure. Ti atom in grey, C atom in black, O atom in azure, Na atom in fuchsia. Na-O bond cutoff has been set to 2.4 Å to highlight the tendency of Na atom to relax though a layer surface.

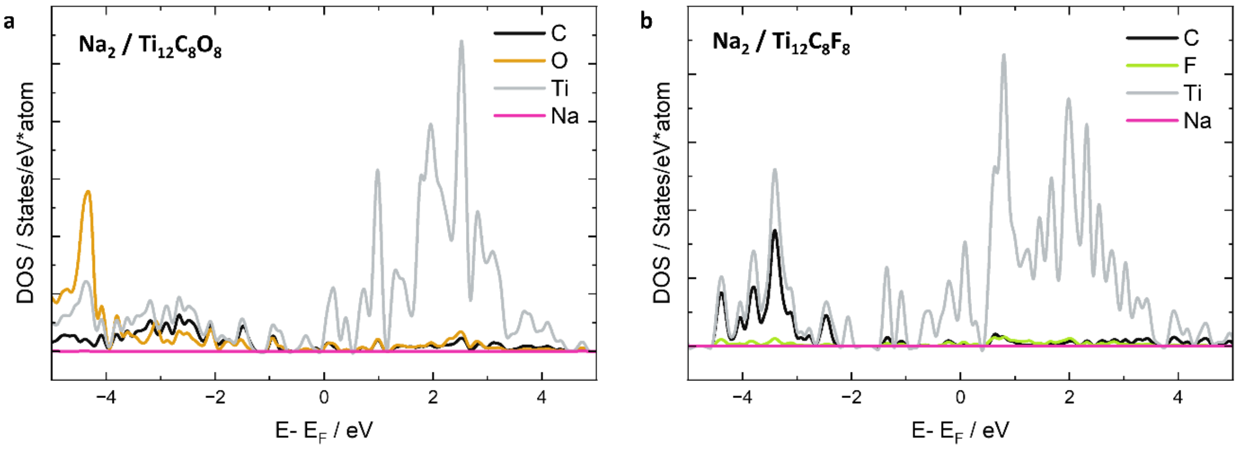

Figure S4. Projected DOS of $Na_2/C_8Ti_{12}O_8$ (left) and $Na_2/C_8Ti_{12}F_8$ (right).

Table S2. Vibrational frequencies of the active normal modes in non-sodiated and sodiated MXenes structures.

**Mode**	**$C_8Ti_{12}O_8$ / cm^{-1}**	**$Na_4C_8Ti_{12}O_8$ / cm^{-1}**	**$C_8Ti_{12}F_8$ / cm^{-1}**	**$Na_4C_8Ti_{12}F_8$/ cm^{-1}**
**1**	747	736	712	677
**2**	650	663	687	727
**3**	616	603	637	610
**4**	585	600	487	317
**5**	545	604	648	686
**6**	503	460	406	285
**7**	428	348	306	301
**8**	358	354	244	236
**9**	337	240	235	130
**10**	238	320	329	350
**11**	215	257	207	317
**12**	110	114	140	78

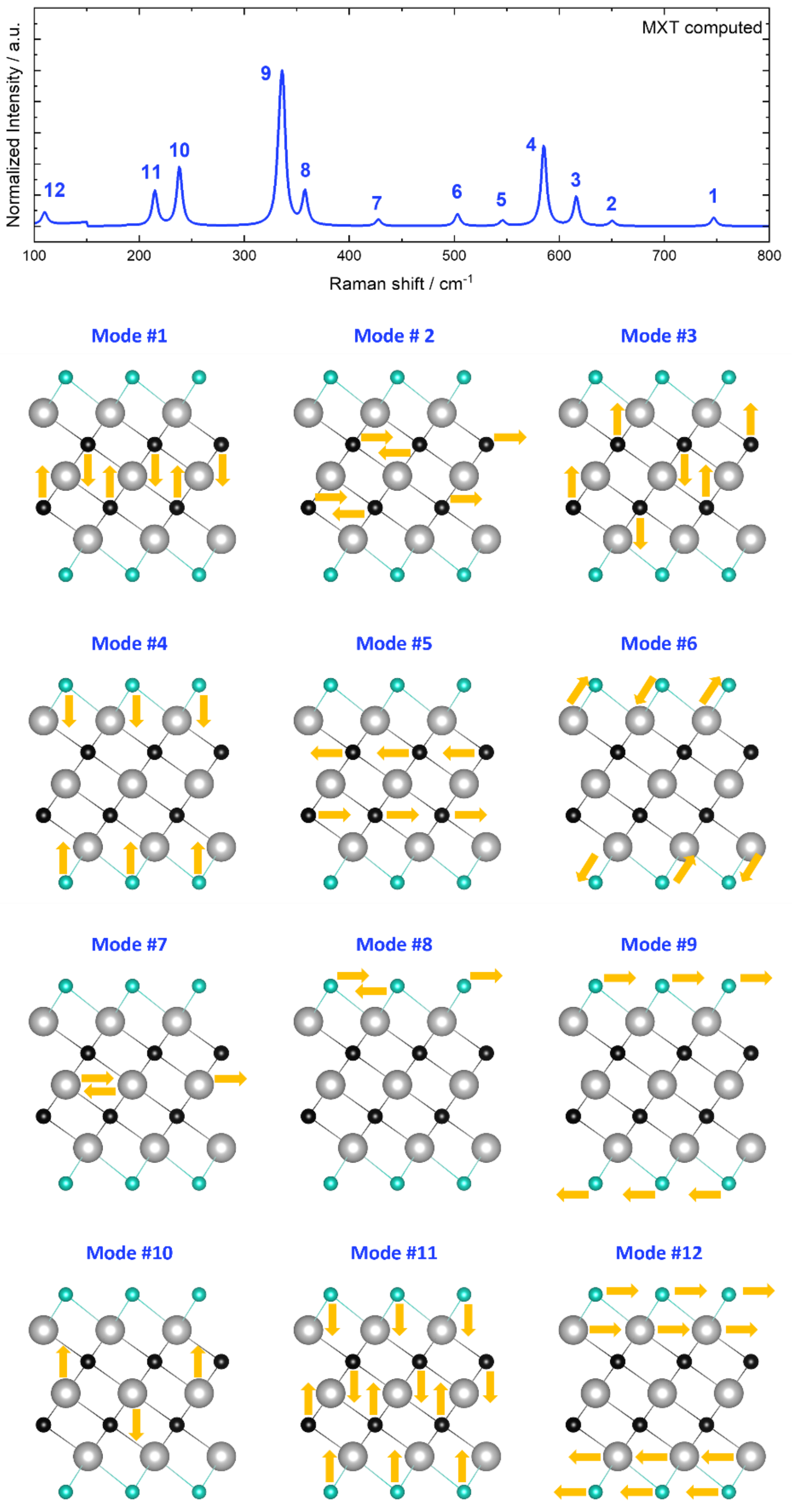

Figure S5. Atomic displacements corresponding to the active Raman vibrations reported in the calculated MXT spectrum obtained for the $Ti_{12}C_8O_8$ composition.

Section 4 – XAS analysis

MCR-ALS method - MCR-ALS strategy (Multivariate Curve Resolution – Alternating Least Squares), combined to PCA (Principal Component Analysis), was the method selected for the analysis of the XAS dataset. This innovative approach has been recently implemented for analysis of batteries investigation since it allows to identify possible hidden intermediates of the reaction and to quantitatively determine the evolution of the species over the explored potential range. [19–21] Detailed theoretical descriptions of the MCR-ALS approach can be found elsewhere. [22,23] Shortly, MCR-ALS allows to decompose a complex data matrix D (m x n) into two simpler matrices, C (m x z) and ST (z x n), containing the concentration profile and the pure spectra of z species, according to the formula: $D = C \cdot ST + E$, where E is the error matrix due to the reconstruction of D. [20] Essentially, this means that it is possible to retrieve the pure spectral components contributing to the dataset, and to quantitively follow their evolution over the potential. This represent a powerful tool in case of analysis of the dataset large enough (tens of spectra) and subtle expected differences; indeed, all the information is achieved through chemometric and mathematical tools, thus allowing to highlight possible elusive phenomena without being influenced by pre-existing assumptions.

Firstly, PCA was used to determine the number of independent components, which resulted to be two for our dataset. Finally, MCR-ALS analysis, obtained by considering the two statistically significant components, was applied to the whole data set, revealing that the whole dataset can be obtained by linear combination fitting of two spectral components, shown by the green and the orange line in Figure 3b-c in the main text.

XAS results – Determination of the oxidation state - Before the operando investigation, XAS spectra were acquired at the Ti K-edge on MXT, and compared to TiO_2 (anatase), Ti_2O_3 and TiO, used as references. The XANES (X-ray absorption near edge structure) region is shown in Figure S6. The pre-edge region of the spectrum, located around 4970 eV, is constituted by two peaks that can be attributed to the Ti 1s → C 2p + Ti 3d (t_{2g} and e_g) hybridized empty orbitals. The main edge (white line, WL), located around 4985 eV, is due to the transition from Ti 1s to Ti 4p unoccupied orbitals above the Fermi level. [24–26] In X-ray absorption spectroscopy, the energy position of the rising edge is indicative of the oxidation state of the material; for MXT the edge energy, marked by a dotted grey line in the figure, is located at ca. 4980 eV and is nearly coincident to that of Ti_2O_3, suggesting a valence state close to Ti^{3+}. Considering a linear dependence between the valence state and the edge energy position for the Ti references, we could estimate an oxidation state of 3.2. However, it should be here noted that this attribution, though convenient for the following discussion, is intrinsically

subjected to a certain error, since different ligands (oxygen and carbon in this case) may lead to a shift in the edge energy position. Moreover, we here note that the MXene phase is obtained by etching of the precursor MAX phase with formula, leaving Ti atoms bonded to O/F terminations. The representation of a single MXene layer with this structure is depicted in Figure 2 in main text. As demonstrated in our previous work, where we employed X-ray diffraction coupled to EXAFS (extended x-ray absorption fine structure) [2], and according to density functional theory (DFT) calculations,[1] the O/F terminations preferentially occupy the position (0,0,z). As determined by neutron diffraction, MXT has a chemical formula close to $Ti_3C_2F_{1.6}O_{0.4}$.[2] Different etching conditions lead to different degree of fluorination, and this may lead to slight shifts in the edge energy position.

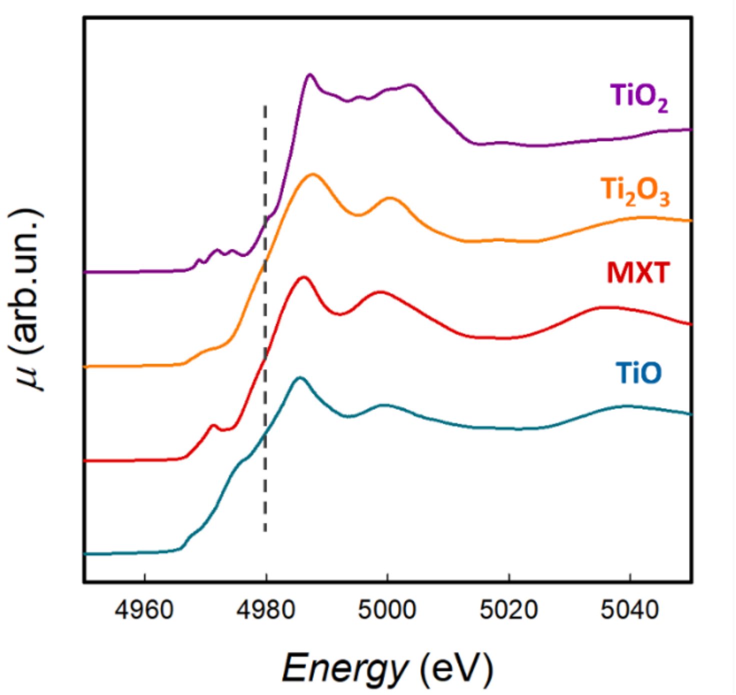

Figure S6. Ex situ XANES spectra at the Ti K-edge of MXTand TiO, TiO_2 and Ti_2O_3, the latter employed as standards.

Table S3. Peak area under *2p* → *3d e_g* peaks of the Ti $L_{2,3}$-edges spectra.

Potential vs Na	Peak area L_2 edge	Peak area L_3 edge
3 V	0.034	0.030
1 V	0.019	0.019
0.1 V	0.017	0.018

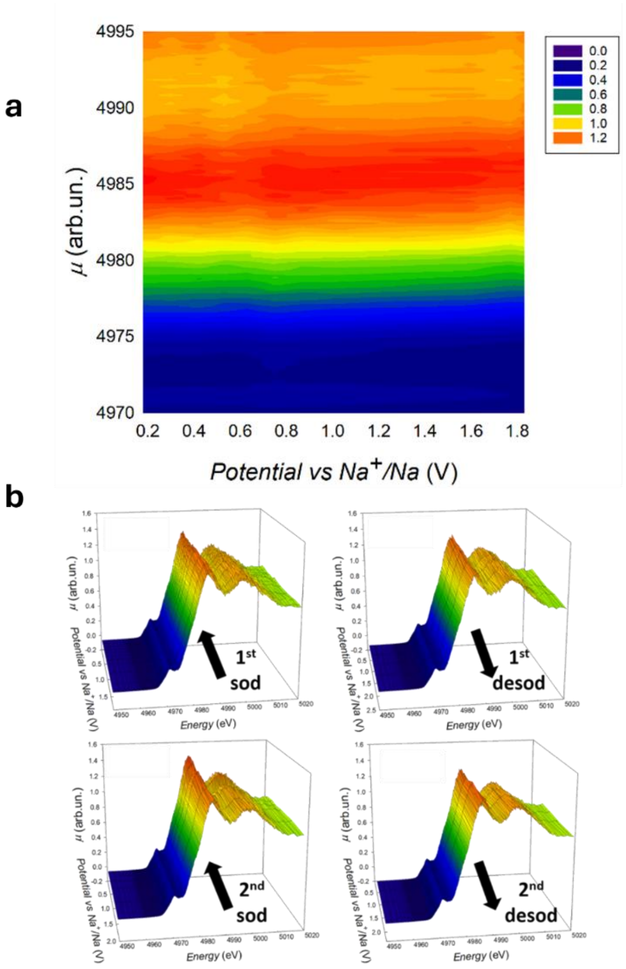

Figure S7. Contour plot of the operando XAS spectra during the first sodiation process (a), line plot of the operando XAS spectra acquired during the first and the second cycle (b).

Section 5 – Raman analysis

Experimental setup and details.

Raman results - In this work, the Raman measurements in operando were collected consecutively for ≈67h equal to 4 cycles of charging and discharging the half-cell vs Na^+/Na with a Mxene-based anode, exploiting setup precutions previously suggested. [27–30] Raman spectroscopy is able to provide information on the surface chemistry, stacking, and quality of $Ti_3C_2T_x$. Vibrations of $Ti_3C_2T_x$ consist of E_g (in-plane) and A_{1g} (out-of-plane) peaks, where the latter are sharper and stronger. The counter map of the whole four cicles in the range 100-800 cm^{-1} are resumed in Figure 4 in the main text and Figure S7.

The agreement between the experimental and calculated spectra are extremally good. The calculated spectrum features a peak at 747 cm^{-1} (coinciding with the peak measured at 733 cm^{-1}), and a series of

peaks between 503 cm^{-1} and 650 cm^{-1}, which coincide with the broad feature observed in the same region of the experimental spectrum. The calculated peaks at 358 and 336 cm^{-1} fits quite closely to the broad peak measured at 367 cm^{-1}. Similarly, the doublet at 215-238 cm^{-1} falls close to the signal measured at 209 cm^{-1}. The only relevant discrepancy concerns the intensity of the soft mode at 110 cm^{-1} (exp. 119 cm^{-1}), which is strongly underestimated. Previous computational results have revealed issues in the region above 700 cm^{-1}, probably due to some approximations in the adopted model (monolayer instead of stacked lamellae).[31]

In the same image Figure S7 are also reported the evolutions during the four charge and discharge cycles of the normalized intensity (solide line) and the Raman shift (dotted line) for the three most intense Raman mode, 1,10 and 12. The spikes in the normalized intensity graphs near +0.1V potential are related to the reduced overall signal of the spectra collected at this cycling condition.

Figure S7 reports the operando Raman measurments and electrochemical profiles for the first four cycles. It is possibile to tentative analize the oscillasion of Raman shifts of the main modes 1, 10, 12 in Figure S7. They range, respectively between 716-735 cm^{-1}, 210-240 cm^{-1}, and 120-150 cm^{-1}. In perfect agreement with DFT results, a blue shift behavior during sodiation is observed for mode 10 and 12 and, vice versa, blue shift for mode 1 during the same kind of electrochemical process. About the mode 1, can be interesting underling the strong similarity between the two extremes, 716 cm^{-1} and 735cm^{-1} of the oscillation range of the Raman shift with those observed in literature when the in-operando Raman measurements have been carried out during sodiation of MXene using acidic electrolyte. [32] Even for this aqueous media, it was observed that the Raman signal of the out-of-plane vibration (A1g) of C-atoms was seen to be within the range between the values for predominant -O- (732 cm^{-1}) and predominant -OH (715 cm^{-1}) terminations implying that the MXene termination chemistry lies between the two extremes during electrochemical charge storage. Thus, there is the possibility that the mechanism of accumulation (proton-assisted) of sodium in MXenes hypothesized by Johnson et al. using an acidic aqueous electrolyte may be the same as in case of organic solvent. This aspect has not been further explored in this work. More complex to interpret is the trend of the intensity of the same Raman signal during the half-cell cycling. Starting from OCV, the profile of the normalized intensity of the mode 1 (716-735 cm^{-1}) shows a maximum during the first sodiation when the potential vs Na^+/Na is +1.4-1.6 V. We can infer that at this potential a positive re-arrangement of MXenes lamellas in the stack favorite the inter-lamella interaction and, so, resonant condition of this Raman mode. A certain component of this increase may be also associate to the formation of SEI during this first sodiation process. After this maximum, during the further potential reduction up to 0.1 V the intensity profile of the mode 1 show a costant reduction. At this potential, this behavior is

globally observed for all the Raman shifts after the complete sodiation of the MXene, except for a slight increase near the Rayleigh scattering region (see green profile in Figure 5c in main text). This trend induces just a modest intensity increase of the mode 12 which has its signal at lowest Raman shift values. The reduction of the intensity of all the Raman shift for the fully sodiated MXene agrees with the results of DFT simulation that confirm a structural relaxation of the MXT framework in this electrochemical condition. From this result, it's possible to infer a strong reduction of the inter-sheet interaction, that also reduce the resonant nature of the most intense Raman modes. Indeed, the same progressive loss of the Raman signals has been also verified by Lioi et al. checking that trend of Raman modes as a function of the thickness of MXene stack. [27] Studying multi-layered MXenes grains ranging from 80 nm to 4 nm, a progressive reduction of Raman signal has been observed with a light increase of Rayleigh signal for ultra-thin samples (4-8 nm), in perfect agreement with our result. However, in our case, the no-complete disappearance of the signal of resonant mode 1 even in conditions of complete sodiation is an indication of the fact that a minimal interaction between the lamellae is preserved and is sufficient to guarantee the reversibility of the sodium accumulation process in the structure.

During the first de-sodiation step, with the potential increase up to +3 V the intensity of the mode 1 shows a maximum at +1.9 V, which is always present even in subsequent charge and discharge cycles (see Figure S7). This maximum, difficult to interpret unambiguously without further analysis, can find its origin in a behavior already observed in other lamellar materials such as MoS_2 [33] and graphene. [34]With increasing thickness (number of lamellas in the stack), the intensities rise roughly linearly up to a specific number of layers, specific for each 2D material (4 layers for MoS_2, 10 layers for graphene), and then decrease for thicker samples (bulk). This anomaly in Raman intensity is explained by considering the multiple reflection of Raman signal inside the layers as well as the interference effect due to the multiple reflection of the incident laser. [34] To interpret the maximum relative to +1.9 V, we can infer that a re-organization of lamellas driven by sodium de-insertion can make switch the optical behavior of MXene grains passing through this optimal condition to enhance the Raman signal.

The lower value (+1.4-1.6V) for this maximum observed during the first sodiation could be attribute to the presence of some irreversible processes during this step first sodiation phase including the formation of the SEI and the insertion into the structure also of that quantity of sodium at which, in literature is associated the "pillaring effect" to spacing the lamellas in an irreversible way. During cycling, this sodium it will never again be extracted from the MXene structure as confirmed in literature. [35]

The results of in operando Raman measurement of MXene (Figure 4b in the main text), exhibits several signals associated with the vibration modes of the layers in the 3D structure. At open-circuit voltage (OCV- red profile in Figure 5c), three prominent peaks are clear at 119, 209, and 734 cm^{-1}, corresponding to the modes listed in Table S2 as numbers 12, 10, and 1. Two of these peaks have strong intensity under Raman set up condition used in this work because, as well described in the literature, the Eg mode at ≈120 cm^{-1} and A_{1g} modes at ≈515 and 720 cm^{-1} (violet band in Figure 4b) are resonantly active under a 785 nm excitation wavelength, being attribute to a transverse plasmonic resonance near 1.5 eV. [27–30] Th 515 cm^{-1} mode is also present in our spectra but is much less intense compared to the other resonant picks.

In addition to these stronger signals and in accordance with DFT simulation, there are two broad bands, respectively, between 320-480 cm^{-1}, a bundle of Raman modes linked to the functional groups on the surface (T_x region)) and 500-700 cm^{-1}, resulting from the overlap of various vibration modes ascribe to C-atoms. During the sodiation process, as the cell potential decreases, all peaks shift, as predicted by the DFT calculations discussed earlier, due to the new interaction that occurs between the layered structure of MXene, sodium ion, and the diffused electron charge on titanium.

Due to the combination and overlap of most vibration modes, this shift is not easily discernible except for the three main peaks, namely, 12 and 10 at higher wavenumbers (blue shift) and 1 at lower wavenumbers (red shift) (see arrows in Figure 4c). The reversibility of these shifts has been monitored during four full GCPL cycles (see Supporting Information - Section 5). In Figure S7 the trends of both the Raman shifts and the normalized intensities of the 3 main vibrational modes (modes 1, 11, 12) are compared as a function of electrode potential profile applied to the half-cell with Ti_3C_2T as working electrode. All the main three modes show a periodicity linked with GCPL profile for both Raman shifts and intensities (normalized). This qualitative trend confirms that these Raman modes are strictly linked with the reversible processes that assure the storage performance of MXenes as anodes for sodium ion batteries.

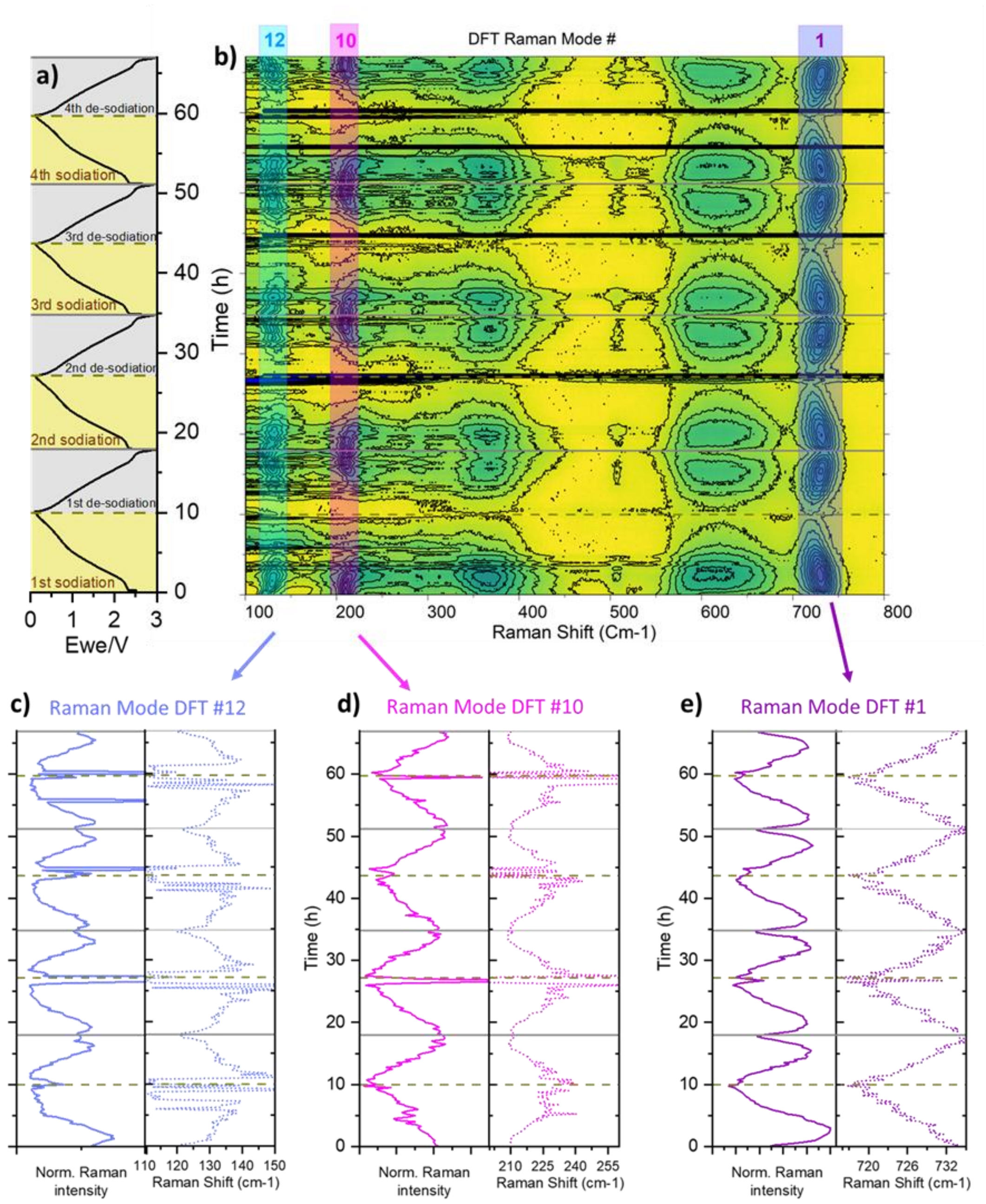

Figure S8. - Galvanostatic cycling with potential limitation (GCPL) profile used for the four electrochemical cycles during the in-operando Raman measurement of MTX sample vs Na^+/Na (a); counter plot of the Raman spectra (after linear background correction) of MTX sample during the four GCPL cycles in the range from +3V to +0.1V (b); evolution during the four charge and discharge cycles of the normalized intensity (solide line) and the Raman shift (dotted line) for the resonant Raman mode 12 (c); evolution during the four charge and discharge cycles of the normalized intensity (solide line) and the Raman shift (dotted line) for the resonant Raman mode 10 (d); evolution during the four charge and discharge cycles of the normalized intensity (solide line) and the Raman shift (dotted line) for the resonant Raman mode 1 (e).

References

(1) Gentile, A.; Ferrara, C.; Tosoni, S.; Balordi, M.; Marchionna, S.; Cernuschi, F.; Kim, M. H.; Lee, H. W.; Ruffo, R. Enhanced Functional Properties of $Ti_3C_2T_x$ MXenes as Negative Electrodes in Sodium-Ion Batteries by Chemical Tuning. *Small Methods* **2020**, *4*, 2000314. https://doi.org/10.1002/smtd.202000314.

(2) Ferrara, C.; Gentile, A.; Marchionna, S.; Quinzeni, I.; Fracchia, M.; Ghigna, P.; Pollastri, S.; Ritter, C.; Vanacore, G. M.; Ruffo, R. The Missing Piece: The Structure of the $Ti_3C_2T_x$ MXene and Its Behavior as Negative Electrode in Sodium Ion Batteries. *Nano Lett* **2021**, *21*, 8290–8297. https://doi.org/10.1021/acs.nanolett.1c02809.

(3) Mizuno, Y.; Sato, K.; Mrinalini, M.; Suzuki, T. S.; Sakka, Y. Fabrication of Textured Ti_3AlC_2 by Spark Plasma Sintering and Their Anisotropic Mechanical Properties. *J Ceram Soc Jap* **2013**, *121*, 366–369. https://doi.org/10.2109/jcersj2.121.366.

(4) Anasori, B.; Gogotsi, Y. *2D Metal Carbides and Nitrides (MXenes)* **2019**, ISBN 978-3-030-19026-2. https://doi.org/10.1007/978-3-030-19026-2.

(5) Gogotsi, Y.; Anasori, B. The Rise of MXenes. *ACS Nano* **2019**, *13* (8), 8491–8494. https://doi.org/10.1021/acsnano.9b06394.

(6) Gentile, A.; Marchionna, S.; Balordi, M.; Pagot, G.; Ferrara, C.; Di Noto, V.; Ruffo, R. Critical Analysis of MXene Production with In-Situ HF Forming Agents for Sustainable Manufacturing. *ChemElectroChem* **2022**, e202200891. https://doi.org/10.1002/celc.202200891.

(7) Lotfi, R.; Naguib, M.; Yilmaz, D. E. A Comparative Study on the Oxidation of Two-Dimensional Ti_3C_2 MXene Structures in Different Environments. *J Mater Chem A* **2018**, *6*, 12733–12743. https://doi.org/10.1039/c8ta01468j.

(8) Naguib, M.; Mochalin, V. N.; Barsoum, M. W.; Gogotsi, Y. 25th Anniversary Article: MXenes: A New Family of Two-Dimensional Materials. *Adv Mater* **2014**, *26*, 992–1005. https://doi.org/10.1002/adma.201304138.

(9) Er, D.; Li, J.; Naguib, M.; Gogotsi, Y.; Shenoy, V. B. Ti_3C_2 MXene as a High Capacity Electrode Material for Metal (Li, Na, K, Ca) Ion Batteries. *ACS Appl Mater Interfaces* **2014**, *6*, 11173–11179. https://doi.org/10.1021/am501144q.

(10) Tang, Q.; Zhou, Z.; Shen, P. Are MXenes Promising Anode Materials for Li Ion Batteries? Computational Studies on Electronic Properties and Li Storage Capability of Ti_3C_2 and $Ti_3C_2X_2$ (X = F, OH) Monolayer. *J Am Chem Soc* **2012**, *134* (40), 16909–16916. https://doi.org/10.1021/ja308463r.

(11) Pianta, N.; Locatelli, D.; Ruffo, R. Cycling Properties of $Na_3V_2(PO_4)_2F_3$ as Positive Material for Sodium-Ion Batteries. *Ionics*, **2021**, *27*, 1853-1860 https://doi.org/10.1007/s11581-021-04015-y/Published.

(12) Kresse, G.; Hafner, J. Ab. Initio Molecular Dynamics for Liquid Metals. *Phys Rev B* **1993**, *47*, 558(R), https://doi.org/10.1103/PhysRevB.47.558.

(13) Perdew, J. P.; Burke, K.; Ernzerhof, M. , *Phys Rev Lett* **1996**, *77*, 3865. https://doi.org/10.1103/PhysRevLett.77.3865

(14) Blochl, P. E. Projector Augmented-wave Method *Phys Rev B* **1994**, *50*, 17953. https://doi.org/10.1103/PhysRevB.50.17953

(15) Grimme, S.; Antony, J.; Ehrlich, S.; Krieg, H. A Consistent and Accurate Ab Initio Parametrization of Density Functional Dispersion Correction (DFT-D) for the 94 Elements H-Pu. *J Chem Phys* **2010**, *132*, 154104. https://doi.org/10.1063/1.3382344.

(16) Baroni, S.; Ab Initio Calculation of the Macroscopic Dielectric Constant in Silicon; *Phys Rev B* **1986**, 33, 7010. https://doi.org/10.1103/PhysRevB.33.7017

(17) Gajdoš, M.; Hummer, K.; Kresse, G.; Furthmüller, J.; Bechstedt, F. Linear Optical Properties in the Projector-Augmented Wave Methodology. *Phys Rev B Condens Matter Mater Phys* **2006**, *73*, 045112. https://doi.org/10.1103/PhysRevB.73.045112.

(18) A. Fonari and S. Stauffer, https://github.com/raman-sc/VASP/, 2013.

(19) Tavani, F.; Fracchia, M.; Pianta, N.; Ghigna, P.; Quartarone, E.; D'Angelo, P. Multivariate Curve Resolution Analysis of Operando XAS Data for the Investigation of the Lithiation Mechanisms in High Entropy Oxides. *Chem Phys Lett* **2020**, *760*, 137968. https://doi.org/10.1016/J.CPLETT.2020.137968.

(20) Fehse, M.; Iadecola, A.; Sougrati, M. T.; Conti, P.; Giorgetti, M.; Stievano, L. Applying Chemometrics to Study Battery Materials: Towards the Comprehensive Analysis of Complex Operando Datasets. *En Stor Mater*, **2019**, *18*, 328–337. https://doi.org/10.1016/j.ensm.2019.02.002.

(21) Conti, P.; Zamponi, S.; Giorgetti, M.; Berrettoni, M.; Smyrl, W. H. Multivariate Curve Resolution Analysis for Interpretation of Dynamic Cu K-Edge X-Ray Absorption Spectroscopy Spectra for a Cu Doped V_2O_5 Lithium Battery. *J. Electrochem. Soc* **2009**, *999*, 3629–3635. https://doi.org/10.1021/ac902865h.

(22) Tauler, R. Multivariate Curve Resolution Applied to Second Order Data. *Chemomet Intell Lab Syst* **1995**, *30*, 133–146. https://doi.org/10.1016/0169-7439(95)00047-X.

(23) De Juan, A.; Tauler, R. Chemometrics Applied to Unravel Multicomponent Processes and Mixtures: Revisiting Latest Trends in Multivariate Resolution. *Anal Chim Acta* **2003**, *500* (1–2), 195–210. https://doi.org/10.1016/S0003-2670(03)00724-4.

(24) Xie, Y.; Naguib, M.; Mochalin, V. N.; Barsoum, M. W.; Gogotsi, Y.; Yu, X.; Nam, K. W.; Yang, X. Q.; Kolesnikov, A. I.; Kent, P. R. C. Role of Surface Structure on Li-Ion Energy Storage Capacity of Two-Dimensional Transition-Metal Carbides. *J Am Chem Soc* **2014**, *136*, 6385–6394. https://doi.org/10.1021/ja501520b.

(25) Magnuson, M.; Näslund, L.-Å. Local Chemical Bonding and Structural Properties in Ti_3AlC_2 MAX Phase and $Ti_3C_2T_x$ MXene Probed by Ti 1s X-Ray Absorption Spectroscopy. *Phys rev Res* **2020**, *2*, *033516.*

(26) Magnuson, M.; Halim, J.; Näslund, L. -Å. Chemical Bonding in Carbide MXene Nanosheets. *J Elec Spec* **2018**, *224*, 27–32.

(27) Lioi, D. B.; Neher, G.; Heckler, J. E.; Back, T.; Mehmood, F.; Nepal, D.; Pachter, R.; Vaia, R.; Kennedy, W. J. Electron-Withdrawing Effect of Native Terminal Groups on the Lattice Structure of $Ti_3C_2T_x$ MXenes Studied by Resonance Raman Scattering: Implications for

Embedding MXenes in Electronic Composites. *ACS Appl Nano Mater* **2019**, *2* (10), 6087–6091. https://doi.org/10.1021/acsanm.9b01194.

(28) Sarycheva, A.; Gogotsi, Y. Raman Spectroscopy Analysis of the Structure and Surface Chemistry of $Ti_3C_2T_X$ MXene. *Chem Mater* **2020**, *32* (8), 3480–3488. https://doi.org/10.1021/acs.chemmater.0c00359.

(29) El-Demellawi, J. K.; Lopatin, S.; Yin, J.; Mohammed, O. F.; Alshareef, H. N. Tunable Multipolar Surface Plasmons in 2D $Ti_3C_2T_x$ MXene Flakes *ACS Nano* **2018**, *12*, 22. https://doi.org/10.1021/acsnano.8b04029.

(30) Chaudhuri, K.; Alhabeb, M.; Wang, Z.; Shalaev, V. M.; Gogotsi, Y.; Boltasseva, A. Highly Broadband Absorber Using Plasmonic Titanium Carbide (MXene). *ACS Photonics* **2018**, *5* (3), 1115–1122. https://doi.org/10.1021/acsphotonics.7b01439.

(31) Hu, T.; Wang, J.; Zhang, H.; Li, Z.; Hu, M.; Wang, X. Vibrational Properties of Ti_3C_2 and $Ti_3C_2T_2$ (T = O, F, OH) Monosheets by First-Principles Calculations: A Comparative Study. *Phys Chem Chem Phys* **2015**, *17* (15), 9997–10003. https://doi.org/10.1039/c4cp05666c.

(32) Johnson, D.; Hansen, K.; Yoo, R.; Djire, A. Elucidating the Charge Storage Mechanism on Ti_3C_2 MXene through In Situ Raman Spectroelectrochemistry. *ChemElectroChem* **2022**, *9*, e202200555. https://doi.org/10.1002/celc.202200555.

(33) Lee, C.; Yan, H.; Brus, L. E.; Heinz, T. F.; Hone, J.; Ryu, S. Anomalous Lattice Vibrations of Single- and Few-Layer MoS2. *ACS Nano* **2010**, *4*, 2695–2700. https://doi.org/10.1021/nn1003937.

(34) Wang, Y. Y.; Ni, Z. H.; Shen, Z. X.; Wang, H. M.; Wu, Y. H. Interference Enhancement of Raman Signal of Graphene. *Appl Phys Lett* **2008**, *92, 043121*. https://doi.org/10.1063/1.2838745.

(35) Kajiyama, S.; Szabova, L.; Sodeyama, K.; Iinuma, H.; Morita, R.; Gotoh, K.; Tateyama, Y.; Okubo, M.; Yamada, A. Sodium-Ion Intercalation Mechanism in MXene Nanosheets. *ACS Nano* **2016**, *10*, 3334–3341. https://doi.org/10.1021/acsnano.5b06958.
